# Role of Liver-Mediated Tolerance in Nanoparticle-Based Tumor Therapy

**DOI:** 10.3390/cells9091985

**Published:** 2020-08-28

**Authors:** Maximiliano L. Cacicedo, Carolina Medina-Montano, Leonard Kaps, Cinja Kappel, Stephan Gehring, Matthias Bros

**Affiliations:** 1 Children’s Hospital, University Medical Center, Langenbeckstrasse 1, 55131 Mainz, Germany; mcaciced@uni-mainz.de (M.L.C.); Stephan.Gehring@uni-mainz.de (S.G.); 2 Department of Dermatology, University Medical Center Mainz, Langenbeckstrasse 1, 55131 Mainz, Germany; gmedinam@students.uni-mainz.de (C.M.-M.); cinkappe@uni-mainz.de (C.K.); 3 Department of Medicine, University Medical Center Mainz, I. Langenbeckstrasse 1, 55131 Mainz, Germany; lenardkaps@googlemail.com

**Keywords:** nano-vaccine, immunotherapy, tumor therapy, liver sinusoidal endothelial cells, Kupffer cells, hepatic stellate cells, tumor-associated macrophages

## Abstract

In the last decades, the use of nanocarriers for immunotherapeutic purposes has gained a lot of attention, especially in the field of tumor therapy. However, most types of nanocarriers accumulate strongly in the liver after systemic application. Due to the default tolerance-promoting role of liver non-parenchymal cells (NPCs), Kupffer cells (KCs), liver sinusoidal endothelial cells (LSECs), and hepatic stellate cells (HSCs), their potential role on the immunological outcome of systemic nano-vaccination approaches for therapy of tumors in the liver and in other organs needs to be considered. Concerning immunological functions, KCs have been the focus until now, but recent studies have elucidated an important role of LSECs and HSCs as well. Therefore, this review aims to summarize current knowledge on the employment of nanocarriers for immunotherapeutic therapy of liver diseases and the overall role of liver NPCs in the context of nano-vaccination approaches. With regard to the latter, we discuss strategies on how to address liver NPCs, aiming to exploit and modulate their immunological properties, and alternatively how to avoid unwanted engagement of nano-vaccines by liver NPCs for tumor therapy.

## 1. Introduction

During the last decades, the development of nanoparticles (NPs) that deliver drugs and biologicals in a cell type-specific manner has received growing interest as a new therapeutic strategy in cancer therapy [1]. Targeting may be an intrinsic property of the NP due to its size and surface properties [2] or can be conferred by conjugated moieties that bind target cell surface receptors, including antibodies, derivatives of natural ligands, and aptamers [3,4].

In case of tumor therapy, NPs may be designed to target tumor cells directly and to deliver cytotoxic drugs or biologicals [5]. More recently, direct targeting of regulatory immune cell types within the tumor microenvironment (TME), comprising tumor-associated macrophages (TAM), myeloid-derived suppressor cells (MDSCs), and regulatory T cells (Treg) that inhibit other immune cells both within the TME and in the periphery, has proven to be an interesting approach [6]. In that case, nano-vaccines may contain a payload, which either acts in a cytotoxic manner or serves to reprogram a regulatory immune cell to counteract tumor tolerance, e.g., by inhibiting the activity of transcription factor STAT (signal transducer and activator of transcription) 3 that promotes expression of protolerogenic proteins like IL-10 [7]. Nano-vaccines aim to exert tumor-specific immune responses by co-delivery of a tumor antigen and an adjuvant to antigen-presenting cells (APCs) like dendritic cells (DCs), which constitute the most important APC population [8,9].

Topical nano-vaccination delivery, e.g., via the skin, may induce predominantly skin-targeting T effector cells. Hence, systemic delivery of a nano-vaccine may be preferable for therapy of metastatic tumors in order to induce T effector cells that may home any organ [10]. However, so far, virtually all types of functionalized NPs have been reported to accumulate in the liver for considerable extent after systemic application [11]. Therefore, unless the liver is the intended target organ of functionalized NP, for example in the case of treatment of liver fibrosis [12] or hepatocellular carcinoma (HCC) [13], the role of the liver as an obstacle in nano-vaccination needs to be elucidated.

This review aims to summarize knowledge on the immunoregulatory activity of liver non-parenchymal cells (NPCs) with a focus on liver sinusoidal endothelial cells (LSECs) and Kupffer cells (KCs) and on their interaction with systemically applied NP. In case of nano-vaccines that are employed to induce, e.g., antitumor responses, unwanted engagement by liver NPCs may result in tolerance-promoting effects. However, NP-based immunotherapeutic strategies may also aim to exploit the default tolerogenic function of liver NPCs for therapy of autoimmune diseases and allergies. Moreover, functionalized NPs have been used to reprogram liver macrophages with regulatory functions towards a proinflammatory state for tumor therapy.

## 2. NPC Populations of the Liver Contribute to its Tolerogenic Activity

The liver constitutes an important tolerance-promoting organ which is conferred by the concerted activity of non-parenchymal liver cell populations [14]. Antigen-specific tolerance in the liver is largely mediated by KCs that constitute the liver-resident macrophage population and by LSECs. Liver DCs have been ascribed an overall tolerogenic role as well, nicely reviewed by Dou et al. [15]. So far, it is well established that NPs engage KCs [16]. However, the role of LSECs in this regard and the consequences of antigen delivery to KCs and LSECs concerning their effect on immune responses have scarcely been addressed yet. Further, hepatic stellate cells (HSC), which also exert important immune-relevant functions, were shown to engage NPs as well [17].

### 2.1. Immunological Role of LSECs

**Figure 1:**
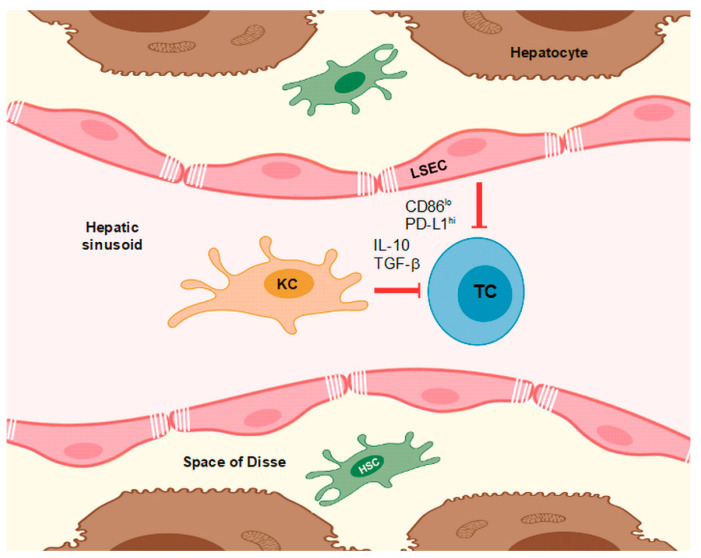
Kupffer cells (KCs) and liver sinusoidal endothelial cells (LSECs) by default confer T cell tolerance by presenting antigens in a non-stimulatory context, characterized by low expression of costimulatory receptors like CD86 but high expression of PD-L1 and by the release of anti-inflammatory cytokines like interleukin (IL)-10 and tumor growth factor (TGF)-β.

LSECs represent about 60% of liver NPCs and are strategically placed lining the hepatic sinusoid [18]. Therefore, LSECs are the first liver cell type that may engage pathogens, immune complexes, and circulating lymphocytes. LSECs possess fenestrations acting as a sieve, which allow the exchange of fluid, solutes, and particles between the blood and the space of Disse [19] (Figure 1). LSECs act as scavengers to clear (potentially dangerous) macromolecules from blood. In addition, as described below, LSECs possess both innate and adaptive immune functions and are highly acknowledged as an important player in liver immunity, including HCC development and progression [18]. Most importantly, under homeostatic conditions, antigen presentation by LSEC leads to tolerance induction in CD8^+^ cells and to the induction of immunosuppressive CD4^+^ regulatory T cells (Treg) [20]. 

### 2.2. Immune-Relevant Properties of LSEC

#### 2.2.1. Phenotype

Phenotypic characterization of LSEC requires combined detection of several surface receptors. LSECs are the only liver-resident cell population that expresses FcγRIIb2 (CD32b) [21], and besides, KCs express the mannose receptor CD206 at high levels [22]. CD45 is highly expressed on periportal LSECs, lowly expressed on midlobular LSECs, and is not apparent on centrilobular LSECs [23,24]. Therefore, differential expression of CD45 in LSECs allows for delineation of their localization within the liver. CD31 serves as a panendothelial cell marker [25]. In addition, LSECs were shown to express von-Willebrand-Factor (VWF), the scavenger receptors Stabilin-1 and -2, vascular endothelial growth factor receptors (VEGFR) 2 and 3, the CD44 homolog lymphatic vessel endothelial receptor (LYVE) 1, and the adhesion receptor CD146 [18,20,23,26]. Accordingly, LSECs can be phenotypically described as CD31^+^CD146^+^ CD32b^+^.

#### 2.2.2. Endocytic Capacity 

LSECs have the highest endocytotic capacity of all human cell types [18]. Their clathrin-dependent endocytic machinery confers efficient uptake of foreign and physiological (waste) products, like connective tissue macromolecules, heparin, lysosomal enzymes, modified proteins and lipoproteins, and soluble IgG complexes [27]. LSECs are equipped with various endocytosis receptors, including scavenger receptors class A (SCARA1-5), B (SCARB1-3), E (SCARE1-2), and H (stabilin-12) [18,23,28]. As mentioned above, LSECs also express different C-type lectin receptors (CLRs). These receptors are predominantly expressed by professional APCs in a population-specific manner [29] and in a CLR-type-specific manner recognizing pathogen-specific glycoproteins [30], endogenous damage-associated molecular patterns derived from dead cells [31], and proteins with a tumor-dependently altered glycosylation pattern [32]. As shown for APCs, both the type of CLR engaged as well as concomitant triggering of other types of receptors, like TLR (Toll-like receptor), determine whether CLR engagement yields stimulatory or inhibitory signaling [33]. 

LSECs express the CLR CD206, which is also apparent on monocytes/macrophages and conventional DCs [34], and L-SIGN (liver/lymph node-specific intercellular adhesion molecule (ICAM)-3 grabbing non-integrin), also known as DC-SIGNR (DC-SIGN-related protein; CD209L), and LSECtin [35]. DC-SIGNR/L-SIGN is a homolog of DC-SIGN (CD209) which is expressed predominantly by cDC and by some macrophage populations [36]. Besides LSECs, macrophages [37], including KC [38] were reported to express LSECtin too. So far, L-SIGN and DC-SIGN were demonstrated to bind largely the same pathogen-associated glycoproteins [39,40], whereas LSECtin was shown to engage only a subset of viruses that bind DC-SIGN/DC-SIGNR [35].

LSECs recognize and endocytose IgG-containing immune complexes via FcγRIIb2 [18,20,23,28]. FcγRIIb is the only Fc receptor that induces inhibitory cell signaling upon engagement [21]. As described by Tanikagi and collegues, stimulation of activating FcγR in endothelial cells, vascular smooth muscle cells, and monocytes/macrophages causes a variety of cellular responses that may contribute to vascular disease pathogenesis [41].

#### 2.2.3. Immune Functions

LSECs express various PRR (pathogen-associated recognition receptors), including TLR2-4, TLR6, TLR8, and TLR9 [42] as well as NOD (nucleotide binding oligomerization domain containing) 1/2 receptors [43], and produce innate cytokines in response to stimulation (e.g., tumor necrosis factor (TNF) α, IL-6, and IL-1β). LSECs exert APC activity during immune surveillance and liver inflammation and predominantly contribute to peripheral immune tolerance [44]. The APC activity of LSECs has been attributed to the constitutive and inducible expression of several surface markers and costimulatory molecules associated with professional APCs, such as major histocompatibility complex (MHC) I and II [45], ICAM (intercellular adhesion molecule) 1 [46], vascular cell adhesion protein (VCAM) 1 [47], CD40, CD80, CD86 [48], L-SIGN, and vascular adhesion protein (VAP) 1 [23]. Of note, LSECs can present antigens of exogenous origin via MHCII to CD4^+^ T cells [49] and via MHCI to CD8^+^ T cells [50], termed cross-presentation. Cross-presentation has also been reported to enable LSECs to present antigens derived from internalized apoptotic tumor cells to CD8^+^ cells leading to T cell tolerance [50]. LSECs express the lectin galectin-1, which is known to bind and to induce apoptosis in activated T cells, resulting in immunological tolerance as well [19]. Of note, besides LSECs, only subpopulations of DCs are capable of cross-presenting antigens derived from internalized material [51]. In the case of LSECs, their tolerogenic phenotype as characterized by programmed death-ligand 1 (PD-L1)^high^ CD80/CD86^low^ expression by default supports induction of CD8^+^ T cell tolerance [52]. Further, lipopolysaccharide-activated LSEC can induce naïve CD4^+^ T cells to produce interferon (IFN)-γ, interleukin (IL)-4, interleukin (IL)-10, and tumor growth factor (TGF)-β but do not induce a stable T helper cell type (Th)1 or Th2 phenotype [53]. Besides, LSECs were reported to diminish the functional activity of Th1 and Th17 via PD-L1 and IL-10 [54]. Moreover, LSECs were shown to induce expression of the anti-inflammatory cytokine IL-10 in Th1 without affecting their IFN-γ production [55]. Thereby, LSECs also contribute to impairing autoreactive CD4^+^ T cells in the periphery. Moreover, as compared to KCs, LSECs are more efficient at inducing differentiation of naïve CD4^+^ T cells to CD25^+^Foxp3^+^ Treg due to their ability to secrete TGF-α and to tether exogenous TGF-β [49,52]. However, stimulation of LSECs with TLR1/2 ligands resulted in a profound increase in their T cell stimulatory activity, accompanied by upregulation of APC surface markers and a release of IL-12 promoting Th1 and cytotoxic T lymphocyte (CTL) induction [56].

#### 2.2.4. Targeting of LSEC with Nanocarriers

Nano-vaccines can be used for treatment of allergies or autoimmune diseases aiming either to promote antigen-specific immune tolerance or to redirect an adaptive immune response. Concerning the former strategy, targeting of LSECs (and KCs) which promote antigen-specific tolerance to self- or foreign antigens by default is an attractive option [57]. Due to inhibitory signaling evoked by triggering FcγRIIb on LSECs, a nano-vaccine designed to induce tolerance may not necessarily require co-delivery of an anti-inflammatory moiety. However, the latter may be necessary to override any potential activating signal induced by addressing a given receptor [58].

The potential of LSECs as a target for immunotherapy has scarcely been issued yet. Finding a way to adjust the NP surface for LSEC targeting is a universal approach to improve the efficacy of NP targeting and drug delivery to endothelial cell types in the liver [4]. LSECs are specialized in the uptake of soluble material and of immune complexes which have a mean diameter of about 40 nm, at a range from 20–150 nm [21]. KCs endocytose material as well but, due to Fc-receptor (FcR) and complement receptor (CR) expression, are also equipped to phagocytose pathogens, including bacteria and fungi [59]. Therefore, nano-vaccines intended to target LSECs should be of a smaller size (≤150 nm) [21].

LSEC-focused NP delivery may require targeting of endocytic surface receptors expressed by this cell population at high density, for example, CD206 [22]. However, this receptor is also expressed by KCs, other macrophages, and conventional DC populations throughout the body [59]. On the contrary, so far, the CLR L-SIGN has been reported as expressed by LSEC only [49]. Nonetheless, since DC-SIGN is homologous to L-SIGN, receptor ligands to be used as LSEC-targeting moieties may also address DCs and macrophages [35]. Engagement of CD206 promotes cross-presentation of derived antigens and may evoke stimulatory cellular signals that enhance APC activity [40]. So far, signaling consequences of triggering CD206 on LSECs as well as L-SIGN have not been studied. LSECs also express FcγRIIb at high extents to internalize immune complexes [60]. This Fc receptor is the only one which transmits an inhibitory signal upon engagement and, therefore, may contribute to the default tolerance-promoting state of LSECs [21]. The potential of LSEC-focused nano-vaccination to reestablish tolerance as required for effective treatment of autoimmune diseases and allergies has also been scarcely analyzed. So far, only one study has been published describing the use of an LSEC-targeting nano-vaccine with therapeutic efficacy in a model of ovalbumine-induced asthma [57].

It has been demonstrated that the default protolerogenic state of LSECs can be overcome by treatment with different stimuli, including TLR ligands. At an activated state, LSECs induce T effector cells [49]. This property might be of general interest with regard to the development of immunotherapeutic strategies for treatment of (liver) cancer, including tumor metastasis in the liver, as well as for treatment of (chronic) infections in this organ [57].

### 2.3. Liver Macrophages Maintain Tolerance under Homeostatic Conditions

#### 2.3.1. KCs Are the Main Macrophage Population under Homeostatic Conditions

KCs have been described as the biggest macrophage population residing in the liver. These resident macrophages are a fundamental pillar for intrahepatic and general innate immunity. Actually, KCs represent around 80% of all tissue macrophages in the body [61]. KCs are mostly located in the liver sinusoids, an ideal place to display their role as sentinels of the immune system. However, recently, it has been described that KCs extend their cell body to the space of Disse where they can interact with HSC and hepatocytes [62]. This kind of interaction is important from a functional point of view as KCs are responsible, e.g., for the transfer of iron from apoptotic red blood cells to hepatocytes [63]. Very recently, a study using single-cell RNA sequencing analyzed the differences between intrahepatic monocyte/macrophage populations [64]. It is well-recognized by now that nonmigratory macrophages derived from embryogenic roots constitute KCs.

**Figure 2:**
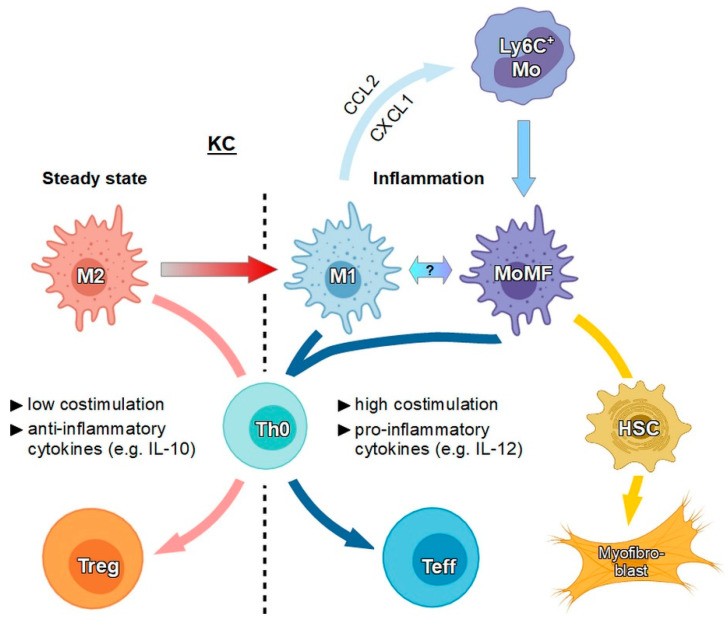
Under steady state conditions, KCs exhibit a protolerogenic M2-like immunophenotype and convert T cells, as shown for naïve CD4+ T cells (Th0), towards Treg due to low co-stimulation and secretion of anti-inflammatory cytokines. In response to stimulation, KCs may acquire an immunogenic M1-like state and may attract Ly6^+^ monocytes (Mo), which then differentiate to monocyte-derived macrophages (MoMFs). The exact relation between KCs and MoMFs is unclear yet. Both immunogenic M1-like KCs and MoMFs induce T effector cells (Teff) since they express costimulatory receptors and pro-inflammatory cytokines at high extents. Furthermore, MoMFs induce trans-differentiation of hepatic stellate cells (HSCs) towards myofibroblasts.

Following injury, there is an influx of monocyte-derived macrophages (MoMFs) into the liver (Figure 2). These macrophages then acquire the KC-specific genetic program [62]. It remains under discussion whether monocyte-derived macrophages in the liver need to be considered as a KC subpopulation [64,65]. MoMFs display pro-inflammatory functions and can be identified by their distinct phenotypic marker profile (HLA-DR^+^CD25^+^CD86^+^) in human liver tissue. MoMFs remove protein complexes and particulate material and apoptotic cells from the blood by phagocytosis [66]. Their function complements LSECs, generating an efficient barrier to avoid penetration by pathogens inside the liver through the portal vein [67]. In comparison, KCs confer immunomodulatory functions and can be identified by markers like CD163, CD206, and CD209 [6]. Besides, KC expresses MARCO (macrophage receptor with collagenous structure) [68]. The expression of MARCO in the tumor microenvironment (TEM) has been related to poor prognosis in different cancers [64].

Although the findings on liver function and immunity in mice usually fit well with human liver, those markers used to identify macrophage populations are different. Hepatic macrophage markers, for example, are commonly CD45^+^F4/80^+^. Of these, MoMFs express markers like CD16, CD32, and CD11c, while KCs express CD206 and CD209 [69,70,71].

#### 2.3.2. KCs Promote Tolerance by Default but Exert Pro-Inflammatory Activity in Case of Liver Inflammation

Macrophages are equipped with various types of danger receptors that enable sensing and recognition of pathogens, such as scavenger receptors, TLR, retinoic acid-inducible gene (RIG)-like receptors (RLR), NOD-like receptors (NLR), and CLR [66]. KCs are equipped with TLR1-9 [72], complement receptors (CR1, CR3, and CR4) [73], and scavenger receptors including class AI/II [74]. 

Under steady state conditions, KCs are of a M2-like phenotype [75], characterized by low expression levels of MHCII, CD80, CD86, and CD40 and by generation of anti-inflammatory IL-10 at considerable extents [76] (see Figure 2). Antigen presentation by KC leads to CD4^+^ T cell arrest and Treg expansion [77] as well as inhibition of antigen-specific T effector cells that have been induced by other APCs [69]. Furthermore, KCs also produce immunomodulatory mediators, such as IL-10, TGF-β, galectin-9, PD-L1, and PD-L2 during hepatitis infection, which suppress antiviral T cell responses [69,78]. In summary, all of these effects elicit tolerogenic immunity [79]. IL-10 release also reduces the production of TNF-α, IL-6, and other cytokines contributing to tolerance [80]. Altogether, KCs are self-renewing, resident, and principally nonmigratory macrophages that serve as sentinels in the liver [65] and serve to promote tolerance by default [81]. Thus, immune responses against harmless antigens like those derived from the diet or from gut microbiota can be avoided [82]. However, under conditions like liver inflammation and fibrosis, KCs may repolarize towards a M1-like proinflammatory phenotype [77]. Above, activation of KC by stimulation of TLR also leads to an increase in CCL2 and chemokine (C-X-C motif) ligand (CXCL) 1 levels, attracting MoMFs [65]. In the case of MoMFs, once a foreign macromolecule is recognized by a danger receptor, a set of inflammatory cytokines like TNF-α, IL-1β, IL-6, IL-12, IL-15, and IL-18 and chemokines like chemokine (C-C motif) ligand (CCL)2–5 [77,83] are released. As a consequence, these mediators induce recruitment of several immune cells to the liver, like neutrophils, natural killer T (NKT) cells, and MoMFs, starting a cascade of immunological responses.

In addition, when KCs are depleted in the course of disease or liver injury, MoMFs have the capacity to regenerate liver macrophages by differentiating to KCs [84]. However, this regenerative situation may lead also to uncontrollable inflammation, which increases the probability of inducing liver damage. Meanwhile, KCs can promote tissue repair but may also induce aberrant tissue repair, resulting in fibrosis and cancer [65,69].

#### 2.3.3. Targeting Hepatic Macrophage Populations with Nanocarriers for Immunotherapy

Liver macrophages work as a sink for all particulate material that circulates within blood. As an immediate consequence, targeting a specific macrophage population within the liver can be a difficult task. In general, NP internalization by hepatic macrophages was shown to be driven by different mechanisms including macropinocytosis, clathrin- as well as caveolin-mediated endocytosis, and additional endocytotic pathways [85]. There are two major cell type-targeting strategies: passive and active targeting. Passive targeting includes the modulation of NP properties, for example, size and surface charge, to increase its probability to reach the specific target cell.

##### Passive Targeting

The uptake of non-functionalized NPs by macrophages has been demonstrated to depend mainly on NP size and on macrophage phenotype. In a recent study, human-derived monocytes were differentiated in vitro towards macrophages using different cytokine cocktails, inducing so-called M1- and M2-like phenotypes, respectively [86]. Regardless of the macrophage phenotype, uptake of gold NP was much higher for NPs with a larger diameter (100 nm) than smaller NPs (15 nm and 60 nm). Notably, for each given type of NP, a big difference in uptake between differentially polarized macrophages was observed: those with a regulatory phenotype (M2 type) showed more than 40% higher uptake than proinflammatory macrophages (M1 type). In agreement, KCs were found to internalize relatively large NPs (˃200 nm Ø) in vivo [85]. An interesting approach to assessing the intrinsic targeting properties of NP in a systemic manner, being mainly performed with liposomes, is the creation of formulation libraries in which several structural changes of NP are tested comparatively, such as changes in the type of phospholipids being used. Cell distribution studies allowed for identification of formulations that preferably accumulate in KC [87]. The preference in NP accumulation by regulatory macrophages has been exploited to induce tolerance against autoimmune diseases. In this regard, NPs loaded with self-antigen were directed to KCs, which in turn presented the antigen and induced T cell tolerance [88,89].

##### Active Targeting

NP functionalized with the sugar moiety mannose intending to target the main mannose binding receptor CD206 (also) expressed by KCs resulted in significantly increased binding to that cell population [78,90,91]. Dual targeting using two different oligosaccharides as ligands for mannose/fucose receptors has proven to induce an accumulation of NPs in murine KCs. Attachment of these ligands, named 4-aminophenyl-α-D-mannopyranoside (APM) and 4-aminophenyl-β-l-fucopyranoside (APF), to liposomes has been used to study the contribution of KC to the accelerated blood clearance phenomenon and for specific depletion of KC [92,93]. However, as mentioned above, CD206 is also expressed by DCs and LSECs, and folate can bind to receptors on normal epithelial cells and tumor cells [94], suggesting that targeting of either receptor may not yield macrophage-specific NP uptake. As an alternative approach, novel synthetic peptides have been developed to target regulatory macrophages in a more exclusive manner. To this end, a peptide library selection approach was followed which allowed identification of a unique targeting ligand for murine M2-type macrophages, named M2pep. M2-type tumor associated macrophages (TAM) have been targeted using M2pep-modified liposomes to deplete them from melanoma [95]. Another study using HCC cells has studied M2pep binding also to TAMs, showing selectivity for M2-type macrophages. The authors also reported about M2pep binding to KCs, though binding to TAM was higher in comparison [96]. In summary, specific targeting to liver macrophage populations has been proven to be very challenging though extremely necessary to achieve different therapeutic objectives such as NP accumulation for specific drug delivery to induce or avoid immune responses or to modulate M1/M2 macrophage balance.

##### Reprogramming of Liver Macrophages

Macrophage plasticity is still a challenging field of study and is of huge interest for therapeutic purposes. Macrophages present in different tissues can modulate their phenotype with dependency on the surrounding environment [97]. Based on this fact, therapeutic strategies are followed, aiming to induce a shift between pro-inflammatory and tolerogenic phenotypes. Concerning the capability of NPs to reprogram hepatic macrophages, silica NPs have been reported to induce the release of TNF-α and IL-1β [98]. In another study, peptide-functionalized gold NPs have been the cause of liver macrophage polarization [99]. In that study, the bioactive tripeptides RGD and GLF were attached to the NP surface. RGD-NP induced a downregulation of both M1 and M2 surface markers. In contrast, GLF-NP upregulated M1 and M2 markers. Other strategies aim to deliver compounds that can activate/inhibit inflammatory pathways or can induce macrophage polarization. Although there is still quite the uncertainty about different molecular interactions involved in the complexity of macrophage functions, some progress has raised interest in this regard. For instance, peroxisome proliferator-activated receptor (PPAR-)γ has emerged as a master regulator for macrophage polarization [100]. Recent studies reported that upregulation of PPAR-γ shifts macrophages polarization from a M1- to a M2-like phenotype. A switch in macrophage polarization was associated with the interaction between PPAR-γ and nuclear factor kappa B (NF-κB) p65 signaling pathways [101]. This report showed that manipulation of PPAR-γ activity can modulate M1/M2 macrophage polarization, having the potential to prevent development of nonalcoholic fatty liver disease (NAFLD). Understanding of these immunomodulatory functions in liver macrophages can lead to the development of novel therapeutic strategies based on macrophage polarization.

Strategies involved in adapting macrophages to acquire a more inflammatory phenotype could be valuable for further development of immunotherapeutic cancer approaches. 

### 2.4. Immunorelevant Functions of HSC

HSCs are mesenchymal cells and compromise about 5–8% of all liver cells [102]. HSCs fulfill a variety of tasks depending on their state as either activated or so-called quiescent HSC. Normally, HSCs are in a quiescent state and constitute the major storage site of vitamin A, secrete extracellular matrix components, and play a role in intercellular communication [103,104]. After their activation, as induced by liver injuries, HSCs transdifferentiate into myofibroblasts [105]. As such, they produce a lot of collagen, which explains their implication in the pathogenesis of liver fibrosis [106]. Besides, activated HSCs also influence the formation and the progression of HCC [107]. In addition, due to their localization near KCs and LSECs in the perisinusoidal space (see Figure 1), HSCs play a role in hepatic immune responses, which qualifies them as a target for immunotherapy.

#### 2.4.1. Immune Functions

Under homeostatic conditions, HSCs primarily contribute to the livers’ immune tolerance, similar to other cell types found within the liver [108]. However, it is known that HSCs also express several TLRs which, when triggered, can induce the release of pro-inflammatory cytokines shaping the livers’ response to injury or infection [109,110,111]. Besides a direct innate immune reaction by HSCs [112,113], this cell population also constitutes an important modulator of immune responses by interacting with immune cells like neutrophils [114], macrophages [115], DCs [112], and LSECs [116] via pro-inflammatory or inhibitory cytokines and trogocytosis, a process in which molecules are exchanged between cells [112]. Furthermore, it has been shown that activated HSCs can act as nonconventional APCs. In this regard, Winau and colleagues demonstrated that HSCs not only are capable of activating CD4^+^ T cells [117,118] but also showed cross-priming capability, resulting in activation of CD8^+^ T cells [119]. These various properties of HSCs to shape the livers’ immune response offer new therapeutic opportunities for liver diseases.

#### 2.4.2. HSC as a Target for Nanocarriers for Immunotherapy

The liver is the main organ in which systemically applied NPs accumulate and are cleared from the body. As outlined above, KCs and LSECs are considered the key players in this regard, but HSCs have also been reported to engulf NPs, albeit to a lesser extent, partly because their location hardly allows any contact with blood-borne particles before these would reach KCs or LSECs [17]. However, active targeting of HSCs by NPs conjugated with moieties that address surface receptors expressed at high density by this cell type may be an effective approach. Popular surface receptors for active targeting of HSCs are the mannose-6-phosphat (M6P) receptor [120], the retinol binding protein (RBP) receptor [121], the platelet-derived growth factor (PDGF) receptor [122], and the collagen type VI receptor [123]. All of these receptors have been targeted successfully in vivo and/or in vitro mainly with liposomal NPs sized between 15 and 400 nm (Ø), aimed to decrease the fibrotic activities of HSCs. Most of the published results about HSCs targeted in the context of NP-mediated immunotherapy are about the improvement of therapy for liver fibrosis, and several nanoparticular systems are already tested in clinical trials [12,124,125]. Although studies about nanocarriers targeting HSCs for the treatment of HCC are missing, by now, this could be a future approach. The potential of HSCs to act as APCs and their ability to cross-talk with other immune cells might open up new possibilities when it comes to the initiation or modulation of an immune response. Depending on the intention, the right type of actively targeted nanocarrier combined with a suitable drug, antigen, and/or adjuvant could vastly improve the therapy of liver fibrosis and presumably the treatment of HCC as well. The antifibrotic or antitumor effects of such types of nanomedicine could even be amplified if several liver cell types, interfering with each other, are targeted at the same time, leading to an effective multi-faceted approach.

Altogether, besides KCs and LSECs, HSCs have also become a target for NP-based (immuno)therapeutic approaches because of their implication in the onset and progression of liver fibrosis and HCC combined with their various immunological functions.

## 3. HCC—Risk Factors and Current Treatment

The liver can be considered an organ with high physical resilience as it possesses a remarkable capacity to regenerate from acute conditions, e.g., hepatectomy or drug-induced-liver injury [126]. Under chronic conditions, its regenerative capacity becomes a two-sided sword. It consists of parenchymal and non-parenchymal cells, while hepatocytes are the most abundant (>80%) and functionally active cells [127]. Albeit debates are still ongoing, there is mounting evidence that HCC derives primarily from transformed hepatic progenitor cells and hepatocytes as a consequence of accumulating genetic mutations and epigenetic alterations [128]. 

### 3.1. Risk Factors for HCC

The chronic component in the development of HCC is underlined by the fact that HCC occurs almost always (approximately 90%) in cirrhotic livers [129]. Cirrhosis can be considered a precancerous condition and represents the most important risk factor of HCC. Accordingly, cirrhotic patients run a yearly risk of approximately 5% for the development of HCC. Cirrhosis is characterized by an excessive accumulation of scare tissue, primarily collagen, in the liver. This severe distortion of the parenchymal and vascular structure represents the end-stage of every chronic liver disease. Other HCC-promoting risk factors are viral hepatitis B (and C), which together account globally for 80% of HCC cases [130,131].

Furthermore, metabolic liver disease has become the most common liver disease in developed countries and an increasing major risk factor for HCC [132,133]. Nonalcoholic fatty liver disease (NAFLD) had the highest population-attributable fraction of 37% for HCC [134]. In contrast to other underlying diseases, HCC occurs frequently in the absence of cirrhosis in NAFLD. A US population-based study of 1500 patients with HCC demonstrated that non-cirrhotic patients with NAFLD-associated HCC had a fivefold risk of having HCC compared to non-cirrhotic patients with hepatitis C virus (HCV)-associated HCC [135]. Diabetes, which displays an increasing incidence in the Western World, is an independent risk factor (2–3-fold) for HCC [136]. Insulin resistance with exacerbated production of reactive oxygen species lead to subclinical chronic hepatic inflammation, being a driver of hepatocarcinogenesis [137,138]. In addition, primary biliary cholangitis, a rare autoimmune disease, has been shown to predispose for HCC development [139]. Further, hemochromatosis, which causes accumulation of iron in inner organs and thereby their dysfunction has been identified as a risk factor for HCC [140]. Alcohol consumption is a common risk factor of HCC in the Western world [141]. Further risk factors are biotoxins such as aflatoxins, which play a minor role in industrialized countries [142].

### 3.2. Current Treatment Options for HCC

Altogether, HCC is the most frequent malignant form of primary liver cancer (annual incidence 7/100,000) and is the second leading cause of cancer-related deaths worldwide, accounting for more than 45,000 deaths per year only in Europe [143]. Management of HCC is complex and depends on the tumor extent, patient’s comorbidities, and the remaining liver function as most treatments risk exacerbating the underlying disease. HCC treatment includes a multidisciplinary team, consisting of hepatologists, visceral surgeons, and interventional radiologists to achieve the best outcomes. Surgical resection is recommended as a curative treatment in HCC patients with respectable disease in an early stage [144,145]. Liver transplantation represents the most definite treatment option when patients meet the Milan criteria [146]. The Milan criteria describe the extent of the disease and take into account quantity, size, gross vascular invasion, and extrahepatic manifestations of the tumors [147]. Percutaneous local ablation by radiofrequency [148] or microwaves as well as transarterial chemoembolization [149] and proton beam are treatment options at an early or intermediate advanced state [148]. However, at advanced stages, systemic treatment remains the last therapeutic option. Sorafenib is a small-molecule multikinase inhibitor that inhibits VEGFR1-3, PDGF receptor-β, and Raf family kinases [150]. It was the first approved drug for first-line systemic treatment, prolonging the median survival of 10.7 months in the sorafenib group vs. 7.9 months in the placebo group [151]. Recently, the multikinase inhibitors levantinib [152] and regorafenib [153] were approved as first- and second-line therapy options after sorafenib treatment, respectively. In 2017 and 2018, the immune checkpoint inhibitors nivolumab [154] and pembrolizumab [155], both PD1-inhibitors, have emerged as second-line therapy, respectively. Recently, ramucirumab, an antiangiogenic VEGFR2 antagonist, expanded the field of second-line therapies and was approved for patients with high serums levels of α-fetoprotein (≥400 ng/mL) and previous treatment with sorafenib [156]. Despite significant progress having been made in systemic therapy of HCC, prognosis is still limited (median survival < 1 year) [130]. Thus, novel therapeutic approaches which are synergistic to established regimes are urgently needed to improve outcomes.

### 3.3. Macrophages Are Key Players in HCC Progression

The tumor biology of HCC can only be fully conceived when considering also the tumor surrounding tissue: the TME [157]. The extracellular matrix represents the non-cellular component of the TME. It contains polysaccharides (e.g., glycosaminoglycan hyaluronic acid) and proteoglycans which are found at high levels in the TME of HCC [158]. Beside the tumor cells, the cellular component of the TME consists of a variety of parenchymal and non-parenchymal cells, including tumor-associated fibroblasts, endothelial cells, adipocytes, and cells of the immune system. The sum of immune cells inside the TME build the tumor immune microenvironment (TIME). There is mounting evidence that the TME and especially the TIME play crucial roles in the development and progression of HCC [157].

KCs and MoMFs play pivotal roles in the development and growth of HCC in the TIME [78]. KCs are liver-resident macrophages, self-renewing, and non-migratory phagocytes and serve as sentinels for liver homeostasis [159]. Upon liver injury, they become activated and excrete inflammatory cytokines (e.g., TNF-α) and chemokines (e.g., CCL2), attracting numerous pro-inflammatory Ly-6C^+^ monocytes from the bone marrow [78]. These inflammatory MoMFs activate HSCs and drive their trans-differentiation into activated myofibroblasts [160]. The latter are the major collagen-producing cells, and a main source of both profibrotic and proangiogenic cytokines (e.g., TGF-β1 and PDGF) in liver fibrogenesis [161].

**Figure 3:**
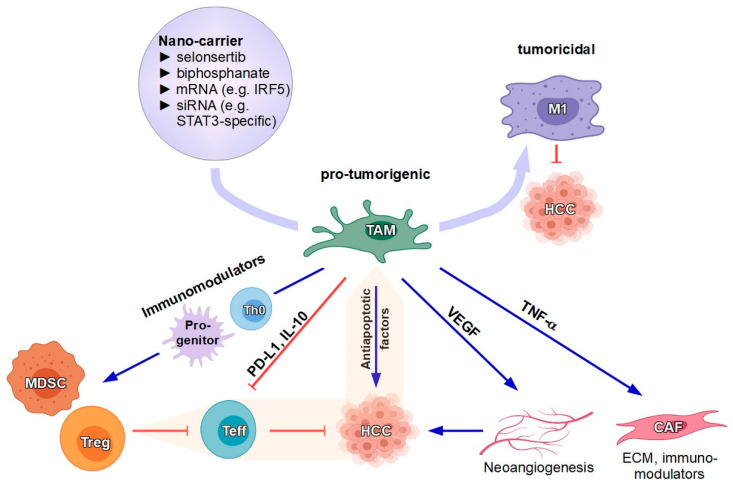
Hepatocellular carcinoma (HCC) generates numerous immunomodulatory soluble factors which govern the differentiation of infiltrating macrophages towards M2-like tumor-associated macrophages (TAMs) and the induction/expansion of myeloid-derived suppressor cells (MDSCs) and Treg and inhibit T effector cells (Teff). TAMs exert overall pro-tumorigenic effects by producing soluble mediators which support tumor progression directly and indirectly via VEGF-dependent neoangiogenesis, and induction of cancer-associated fibroblasts (CAFs) that also shape the tumor microenvironment (TME). Similar to HCC, TAMs also inhibit infiltrating Teff and promote MDSCs as well as Treg that also inhibit antitumor responses. Due to their crucial role, reprogramming of TAMs towards M1-like macrophages with tumoricidal activity has been evaluated using nanocarriers that deliver nucleic acid-based therapeutics. Moreover, drugs like selonsertib and bisphosphonate, previously shown to repolarize TAMs, are suitable payloads for TAM-targeting nanocarriers, thereby minimizing cytotoxicity.

Since both KCs and MoMFs possess high plasticity, TAMs are thought to derive from these two distinct macrophage populations [78]. As high numbers of TAMs are regularly observed in resections or explants of patients with HCC, they are supposed to promote development and progress of HCC [162]. This assumption is further supported by the fact that TAM numbers correlate with HCC progression and poor survival [163]. TAMs were also found to express PD-L1, which suppresses anti-tumoral CTL responses [164,165] (Figure 3). Furthermore, TAMs provide soluble factors that favor tumor cell proliferation, inhibit apoptosis of cancer cells and promote angiogenesis [157], and induce the conversion of fibroblasts towards cancer-associated fibroblasts (CAFs) [166], which in turn via modulation of the ECM and the production of numerous immunomodulatory soluble mediators shape the TME [167]. TAMs retain their plasticity and can switch their phenotype towards “antifibrotic” and putatively “anti-tumor” macrophages [168]. This phenotype is characterized by low expression of Ly-6C in mice and high expression of anti-inflammatory mediators (e.g., HGF and IL-10) and matrix (degrading) metalloproteinases (e.g., MMP-9, MMP-12, and MMP-13) [169]. Thus, a drug-induced phenotypic switch towards “good-natured” macrophages is an appealing concept and has gained increasing attention in basic and drug translational research in the last decade.

### 3.4. Targeting of TAMs with NPs for Tumor Therapy

TAMs express rather a tolerogenic phenotype and thus provoke tumor progression and metastasis [170]. In this sense, it has been of great interest to induce TAM polarization towards a proinflammatory state that can elicit immune responses and tumor regression. TAMs can be targeted by nanoparticle-based drug delivery [13]. Nanocarriers are ideal for this purpose for three reasons. First, TAMs as phagocytes have a high scavenging capacity and efficiently engulf foreign particles, including NPs, by passive targeting [171]. Second, after intravenous injection in mice, the majority of nanocarriers like nanohydrogel particles (approximately 50 nm Ø) [172,173], hard-shell microbubbles (approximately 2 nm Ø), liposomes (approximately 2 nm Ø), and polymers (approximately 10 nm Ø) [90] accumulate efficiently in the liver and arrive in close proximity to liver macrophages. Third, cell-specific active targeting of NP may enhance their uptake by TAMs. TAMs express CD206, which efficiently binds mannose residues at high extents [174]. Mannose-functionalized nanohydrogel particles (ManNPs) loaded with colony stimulating factor (CSF)-1 receptor small interfering RNA (siRNA) demonstrated a robust knockdown of CSF-1 in CD206 overexpressing primary macrophages in vitro, while CD206-negative macrophages were not affected [173]. Thus, ManNPs represent a promising platform for cell type-specific delivery of siRNA to profibrotic macrophages which share characteristics with TAMs.

A recent study reported the use of polymeric NPs engineered to deliver mRNA-encoded modulatory proteins. Delivery of mRNA species that encoded interferon regulatory factor (IRF) 5 and IκB kinase (IKK)β, which activates IRF5 [175], was intended to cause a shift of TAMs towards a pro-inflammatory and cytotoxic M1-like phenotype [176]. In line, in in vivo models of advanced-stage ovarian cancer, metastatic melanoma, and glioblastoma, a reduced density of TAM in tumor lesions and concomitantly a marked increase in inflammatory myeloid cells with M1-type transcriptional profiles was observed. Even though this approach has not been tested for hepatic macrophages, the efficacy of this strategy proves that NP may be employed, e.g., for tumor therapy to repolarize macrophages with regulatory function to exert pro-inflammatory effects.

siRNA are double-stranded noncoding RNA oligos (20–25 base pairs) that sequence-specifically hybridize with their target mRNA to induce its degradation and to thereby diminish its half-life and translation [177]. RNA inference by siRNA allows transient silencing of virtually any gene, offering a huge value for therapeutic applications. It can be envisioned that NPs loaded with siRNA which target relevant pathways of TAMs may induce a phenotype switch or apoptosis of TAMs [178]. Cell type-specific delivery of therapeutic siRNA with functionalized carriers (e.g., ManNPs) to TAMs could improve efficacy and could avoid off-target effects in non-targeted cell types. For example, in a human tumor xenograft model, lipid NP loaded with siRNA specific for the M2-promoting transcription factor STAT (signal transducer and activator of transcription) 3 repolarized TAMs towards a M1-like immunophenotype and therefore reverted their pro-tumoral effects [179].

Hepatic macrophages and hepatocytes share a set of intracellular inflammatory signaling pathways (e.g., NF-κB, apoptosis signal-regulating kinase 1 (ASK-1), c-Jun N-terminal kinase, and p38) [180]. It is conceivable that specific inhibitors of inflammatory signaling like the ASK-1 inhibitor selonsertib have effects not only on hepatocyte metabolism but also on macrophage activation [78,181]. Encapsulation of this small-molecule drug in NPs could enhance the effect on TAMs. Further, bisphosphonates are anti-resorptive agents used in the clinic for osteoporosis [182] and complications of bone metastasis [183]. There is evidence that bisphosphonates also have an effect on the phenotype of macrophages, shifting it from pro-tumoral to tumoricidal [184]. Since bisphosphonates are largely excreted by the urinary tract und rapidly bind to bones upon intravenous administration, their encapsulation by biocompatible carriers could be of interest to target TAMs.

Altogether, TAMs are immunosuppressive cells in the TIME of HCC and were identified as a crucial cell population to fuel tumor development and growth. Therapeutic targeting of TAMs seems promising and might be achieved either by small-molecule or siRNA-based drugs encapsulated in NPs.

## 4. Conclusions

**Figure 4:**
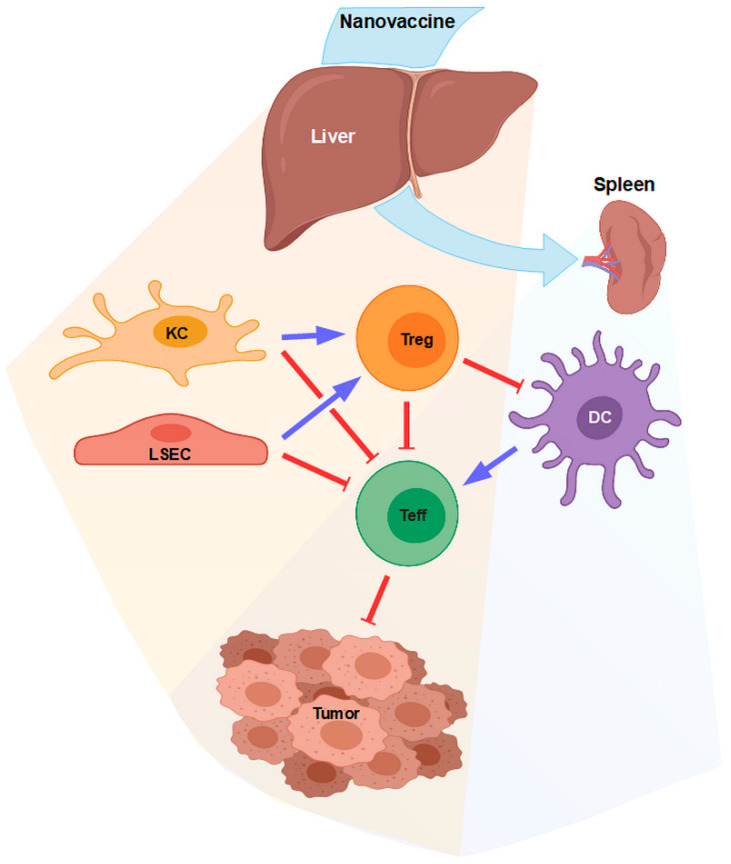
Systemically applied nano-vaccines often accumulate in the liver and reach secondary lymphoid organs only at low extents. In liver, nano-vaccines may be internalized largely by KCs and LSECs, which at default state promote Treg induction and could inhibit tumor-specific T effector cells induced by dendritic cells (DCs) in lymphoid organs. To avoid this outcome, nano-vaccines may either be designed to contain adjuvants that activate KCs/LSECs or to avoid unwanted uptake by liver non-parenchymal cells (NPCs).

Liver NPCs are equipped with numerous receptors to internalize material and, by default, to confer tolerance [76]. Consequently, liver NPCs also strongly bind systemically applied NPs which, on the one hand, can be exploited for direct targeting of KCs or LSECs, e.g., to induce antigen-specific tolerance [57], but, on the other hand, constitutes an unwanted outcome in the case of NPs applied to evoke immune responses in secondary lymphoid organs. With regard to the latter, it is not clear yet whether LSECs and KCs that internalize nano-vaccines aimed to induce antitumor T cell responses may promote tumor antigen-specific tolerance as depicted in Figure 4 and therefore to counteract vaccine-induced adaptive immune reactions. As a perspective, nano-vaccines may be designed to co-deliver adjuvants that activate not only APCs in secondary lymphoid organs but also LSECs [185] and KCs [186] to promote the establishment of effector T cell responses throughout the body. However, it needs to be taken into account that hyperactivated liver NPCs, for example, M1-type KCs, may also cause inflammation and tissue damage [101,187]. Alternatively, nano-vaccines aimed to induce adaptive immune responses in secondary lymphoid organs may be generated in such a manner that unwanted binding and uptake by liver NPC is largely avoided. For this, nano-vaccines should not be decorated with APC-targeting moieties that are also recognized by surface receptors expressed by either liver immune cell population (e.g., CD206 [34]). In general, cellular interaction of NPs and NP-induced alterations of the cellular immunophenotype should be assessed first in vitro, e.g., by using in parallel assays murine liver NPCs and spleen cells, also taking into account that a serum-dependently formed protein corona may strongly alter the targeting properties of NPs [188].

## Abbreviations

APC	antigen presenting cell
APF	4-aminophenyl-β-l-fucopyranoside
APM	4-aminophenyl-α-d-mannopyranoside
ASK-1	apoptosis signal-regulating kinase 1
CAF	cancer-associated fibroblasts
CLR	C-type lectin receptor
CCL	chemokine (C-C motif) ligand
CLR	C-type lectin receptor
CR	complement receptor
CSF-1	colony stimulating factor 1
CTL	cytotoxic T lymphocyte
CXCL	chemokine (C-X-C motif) ligand
DC	dendritic cell
DC-SIGNR	DC-SIGN-related protein
FcR	Fc receptor
HCC	hepatocellular carcinoma
HSC	hepatic stellate cell
ICAM	intercellular adhesion molecule
IFN	interferon
IL	interleukin
IRF	interferon regulatory factor
KC	Kupffer cell
L-SIGN	liver/lymph node-specific intercellular adhesion molecule [ICAM]-3 grabbing non-integrin
LSEC	liver sinusoidal endothelial cells
LYVE-1	lymphatic vessel endothelial receptor 1
M6P	mannose-6-phosphat
ManNP	mannose-functionalized nanohydrogel particles
MARCO	macrophage receptor with collagenous structure
MDSC	myeloid-derived suppressor cell
MHC	major histocompatibility complex
MMP	matrix metalloproteinase
MoMF	monocyte-derived macrophages
NAFLD	non-alcoholic fatty liver disease
NF-κB	nuclear factor kappa B
NKT	natural killer T cell
NOD	nucleotide binding oligomerization domain containing
NPC	non-parenchymal cell
NP	nanoparticle
PDGF	platelet-derived growth factor
PD-L1	programmed death-ligand 1
PPAR	peroxisome proliferator activated receptor
RBP	retinol binding protein
PRR	pathogen-associated molecular pattern
RIG	retinoic acid-inducible gene
RLR	RIG-like receptor
SCAR	scavenger receptor
siRNA	small interfering RNA
STAT	signal transducer and activator of transcription
TAM	tumor-associated macrophage
TEM	tumor microenvironment
TIME	tumor immune microenvironment
TGF	tumor growth factor
Th	T helper cell
TLR	Toll-like receptor
Treg	regulatory T cell
VAP	vascular adhesion protein
VCAM	vascular cell adhesion protein
VEGFR	vascular endothelial growth factor
VWF	von-Willebrand-Factor
